# Bacterial Metabolites in the Plasma of Type 1 Diabetes Patients: Acetate Levels Are Elevated and Correlate with Glycated Haemoglobin and Para-Cresol Is Associated with Liver Disturbances and Hypertension

**DOI:** 10.3390/ijms27020989

**Published:** 2026-01-19

**Authors:** Inés Jiménez-Varas, Martín Cuesta-Hernández, María Inmaculada Domínguez-Mozo, Iván Pérez-Gutiérrez, Stefano Ruberto, Esther Palacios, Ana Moreno-Blanco, Rosa Del Campo, María Ángel García-Martínez, Roberto Álvarez-Lafuente

**Affiliations:** 1 Servicio de Endocrinología, Hospital Clínico San Carlos, 28040 Madrid, Spain; ines.jimenez@salud.madrid.org (I.J.-V.); cuestamartintutor@gmail.com (M.C.-H.); 2 Grupo de Investigación de Factores Ambientales en Enfermedades Degenerativas, Instituto de Investigación Sanitaria del Hospital Clínico San Carlos, 28040 Madrid, Spain; ivaper03@ucm.es (I.P.-G.); stefano.palermo.externo@salud.madrid.org (S.R.); garcia.angel23@gmail.com (M.Á.G.-M.); ralvarezlafuente@yahoo.es (R.Á.-L.); 3 Servicio de Microbiología, Instituto Ramón y Cajal de Investigación Sanitaria, 28034 Madrid, Spain; esthepalgo@gmail.com (E.P.); anamorenoblanco1993@gmail.com (A.M.-B.); rosacampo@yahoo.com (R.D.C.)

**Keywords:** acetate, para-cresol, glycated haemoglobin, short-chain fatty acids, medium-chain fatty acids, type 1 diabetes

## Abstract

Type 1 Diabetes (T1D) is thought to result from the interaction of genetic and environmental factors, with different studies highlighting a potential role for the gut microbiota and its metabolites in modulating immune responses and disease development. We hypothesized that patients with T1D exhibited altered levels of circulating bacterial metabolites compared with healthy controls (HC), and that these metabolite profiles were associated with key demographic, clinical, and analytical features of the disease. A total of 91 T1D patients and 58 HC were recruited. Plasma samples were collected and analyzed with gas chromatography coupled to mass spectrometry for the detection of the metabolites: short-chain fatty acids (SCFAs: acetate [AA], propionate [PA], isobutyrate [IBA], butyrate [BA], isovalerate [IVA], valerate [VA], and methyl valerate [MVA]), medium-chain fatty acids (MCFAs: hexanoate [HxA] and heptanoate [HpA]) and para-cresol (p-cresol). We also calculated the ratios between the different SCFAs with AA. T1D patients showed significantly higher circulating AA levels than HC, along with reduced PA/AA and IBA/AA ratios, indicating an altered SCFA profile. SCFA diversity was lower in T1D patients, with reduced detection of BA, and total SCFA levels were increased mainly due to elevated AA. AA levels were higher and SCFA ratios lower in women with T1D compared with healthy women, while p-cresol levels were higher in men with T1D than in healthy men. In T1D patients, AA levels positively correlated with HbA1c, whereas PA/AA, IBA/AA, and BA/AA ratios showed negative correlations, particularly in women. MV/AA and non-AA/AA ratios were inversely associated with glucose levels, again, mainly in women. p-cresol levels correlated positively with age and ferritin and were higher in T1D patients with liver dysfunction or hypertension. Therefore, we can conclude that T1D is associated with a marked alteration in circulating gut-derived metabolites, characterized by increased AA levels, particularly in women, and an imbalance in SCFA ratios that correlates with glycemic control. These findings, together with the associations observed for p-cresol and metabolic comorbidities, support a role for the gut microbiota–host metabolic axis in T1D.

## 1. Introduction

Type 1 Diabetes (T1D) is an autoimmune condition where the body’s immune system mistakenly attacks and destroys insulin-producing beta cells in the pancreas, leading to little or no insulin production [1]. Insulin is essential for regulating blood sugar levels, and without it, individuals experience hyperglycemia, which can be life-threatening if not managed properly. T1D is typically diagnosed in children and young adults, although it can develop at any age [1]. Management of T1D involves lifelong insulin therapy, regular blood glucose monitoring, a balanced diet, and physical activity to prevent complications like cardiovascular disease, neuropathy, and kidney damage [2].

Although the precise etiology of T1D is not fully understood, extensive research indicates that both genetic and environmental factors contribute to its development. Genetic predisposition is particularly important, with certain genes, especially those within the human leukocyte antigen (HLA) region, conferring increased susceptibility. In addition, genome-wide association studies (GWAS) have identified more than 50 other loci that influence T1D risk [3,4]. However, environmental factors, such as viral infections (e.g., enteroviruses), early diet, and even geographic location, are also thought to trigger the onset of T1D in genetically susceptible individuals [5]. Viruses like the Coxsackievirus B and rubella have been implicated in initiating the autoimmune response that leads to the destruction of beta cells [6]. Other environmental influences, including exposure to certain chemicals or a lack of vitamin D, may also play a role in modulating immune function and increasing the risk of T1D [7]. These findings highlight the complex interaction between genetics and environmental exposures in the pathogenesis of T1D.

In recent years, different studies have explored the potential role of gut microbiota and its metabolites in modulating immune responses and influencing the development of autoimmune diseases like T1D [8]. Short-chain fatty acids (SCFAs), medium-chain fatty acids (MCFAs), and para-cresol (p-cresol) are some of these metabolites, which are in most cases of exclusive bacterial production. Butyrate (BA), a SCFA, has been shown to have anti-inflammatory effects, potentially helping to regulate the immune system and protect against autoimmune diseases like T1D [9]. Studies in feces have suggested that an altered gut microbiome may be associated with an increased risk of T1D [10]. SCFAs may promote the generation of regulatory T cells, which are essential in preventing the immune system from attacking the body’s own tissues, including beta cells [11]. As such, modulation of the gut microbiota and its metabolites may offer a potential avenue for preventing or managing T1D in the future.

Thus, we performed the current study with the aim of analyzing the levels of circulating SCFAs, MCFAs, and p-cresol in a cohort of T1D patients and comparing them with those in a cohort of healthy controls (HC). In addition, based in previous results of our group in other autoimmune disease like multiple sclerosis [12,13,14], we also analyzed the ratios between AA and the other SCFAs since AA, unlike the other SCFAs, seems to have a pro-inflammatory role [15,16,17]. Furthermore, we also tried to correlate the levels of the SCFAs and their ratios and the levels of MCFAs and p-cresol with different demographic, clinical, and analytical variables collected from the T1D patients.

## 2. Results

### 2.1. Gut Metabolite Levels and Ratios in T1D Patients and HC

**Figure 1:**
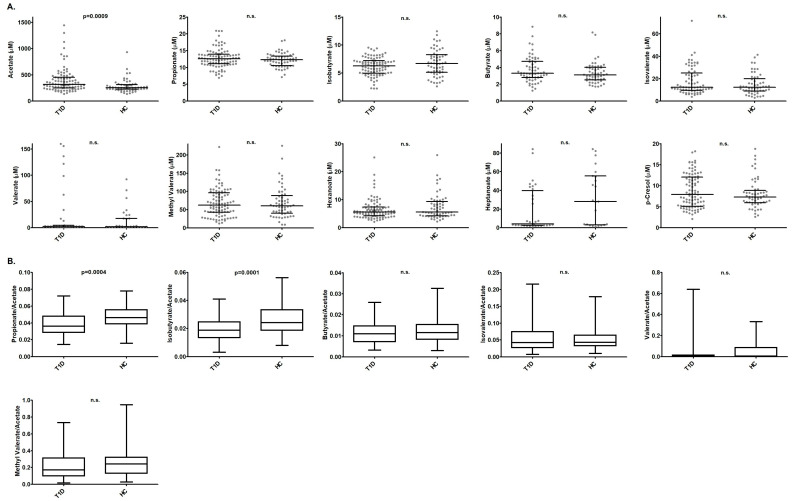
(**A**) Comparisons of gut metabolite concentrations between T1D patients and HC. (**B**) Comparisons of the different SCFA/AA ratios between T1D patients and HC. *p*-values were calculated using Student’s *t*-test or the Mann–Whitney U test in case the values of the SCFA or the ratios did not fit a normal distribution. Only significant *p*-values after Bonferroni correction are shown (*p* < 0.005). n.s.: not significant.

After Bonferroni correction for multiple comparisons, we found that AA levels were significantly higher in T1D patients than in HC (*p* = 0.0009) (Figure 1A). In relation to the SCFA ratios, PA/AA and IBA/AA ratios were significantly higher in HC than in T1D patients (*p* = 0.0004, *p* = 0.0001, respectively) (Figure 1B).

### 2.2. Diversity of SCFAs in T1D Patients and HC

While we detected a median of 7/7 different SCFA in HC, only 6/7 SCFA were detected in T1D patients (*p* = 0.004). Thus, BA was only detected in 61/91 (67.4%) T1D patients vs. 52/58 (89.7%) HC (*p* = 0.003); IVA was detected in 70/91 (76.1%) T1D patients vs. 54/58 (93.1%) HC (*p* = 0.02) but difference was not statistically significant after Bonferroni correction.

A significantly higher total amount of SCFAs was found in T1D patients than in HC (median values: 438.0 μg/mL vs. 365.6 μg/mL, respectively; *p* = 0.003). However, when we excluded the AA, there was not any significant difference between T1D patients and HC (median values: 103.7 μg/mL vs. 103.5 μg/mL, respectively). Furthermore, as higher is the percentage of AA respecting the whole amount of SCFAs, as lower is the number of the different SCFAs detected: the percentage of AA in HC with ≥6 SCFAs was 70.9% vs. 87.3% in HC with <5 SCFA (*p* = 0.01), and similarly, 76.4% in T1D patients with ≥6 SCFA vs. 83.4% in T1D with <5 SCFA (*p* = 0.04).

### 2.3. Association with Sex and Age of the Gut Metabolite Levels and Ratios in T1D Patients and HC

We found that PA/AA, IB/AA, BA/AA, and MV/AA ratios and p-cresol levels were significantly higher in HC women (HCw) than in HC men (HCm) (*p* = 0.009, *p* = 0.03, *p* = 0.04, *p* = 0.03 and *p* = 0.04, respectively), while no differences were found between T1D women (T1Dw) and T1D men (T1Dm) (Table 1). When we compared HCw vs. T1Dw, we found almost the same significant differences as those for the whole population: higher levels of AA (*p* = 0.003) and lower PA/AA, IBA/AA, and BA/AA ratios (*p* = 0.00008, *p* = 0.0002 and *p* = 0.041, respectively) in T1Dw than in HCw. T1Dm showed higher levels of p-cresol than HCm (*p* = 0.022) (Table 1). Regarding age, we did not find any correlation in HC; however, in T1D patients, a positive correlation between p-cresol and age was found (*p* = 0.0001; r = 0.401), present in both women (*p* = 0.005; r = 0.387) and men (*p* = 0.003; r = 0.464).

### 2.4. Gut Metabolite Levels and Ratios in T1D Patients and Their Possible Association with Clinical and Analytical Variables

**Figure 2:**
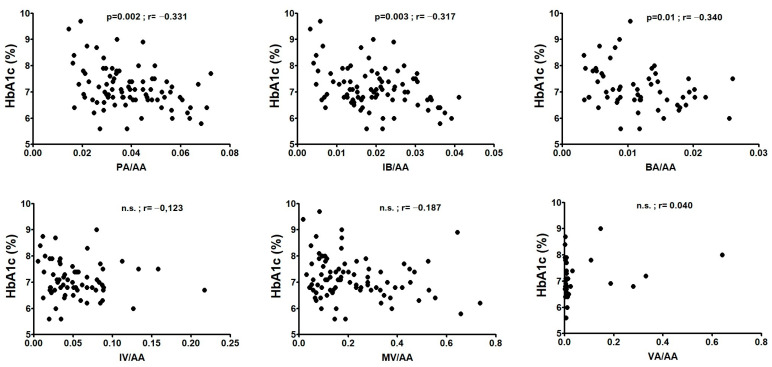
Correlations between SCFA ratios with the percentage of glycated haemoglobin (HbA1c) in T1D patients. Correlations were assessed by using Spearman’s rank correlation coefficient (r); n.s.: not significant.

Glycated haemoglobin (HbA1c) was positively correlated with AA levels (*p* = 0.0001; r = 0.406), while a significant negative correlation with a rSpearman < −0.3 was found for PA/AA, IBA/AA, and BA/AA ratios, as we can see in Figure 2. When we analyzed both sexes, while correlations in T1D women where similar to those of the whole population: AA levels (*p* = 0.001; r = 0.463), PA/AA (*p* = 0.003; r = −0.419), and IBA/AA (*p* = 0.002; r = −0.451), only AA levels (*p* = 0.04; r = 0.334) correlated with HbA1c in T1D men. The ratio between the total amount of SCFA, excluding AA and AA levels, was inversely correlated with HbA1c in T1D women (*p* = 0.006; r = −0.393).

MV/AA ratio negatively correlated with levels of glucose of T1D patients (*p* = 0.002; r = −0.334). This association was also found in T1D women (*p* = 0.0005; r = −0.473) but not in T1D men. Furthermore, the ratio between the total amount of SCFA excluding AA and AA levels was inversely correlated with glucose levels (*p* = 0.002; r = −0.335); this association was also found in T1D women (*p* = 0.002; r = −0.438) but not in T1D men.

Other significant correlation with continuous variables and a rSpearman < −0.3 or >0.3 was between p-cresol and Ferritin levels (*p* = 0.04; r = 0.362).

Regarding the categorical variables, p-cresol levels were lower in T1D patients with a normal liver profile than in T1D patients with a not normal liver profile (7.5 μM vs. 13.0 μM, respectively; *p* = 0.002), and in T1D patients without high blood pressure than in T1D patients with high blood pressure (6.7 μM vs. 10.6 μM, respectively; *p* = 0.005).

## 3. Discussion

Here, we described for the first time, at least to the best of our knowledge, that serological AA levels were significantly increased in T1D patients, mainly in T1D women. Furthermore, a positive correlation between AA and HbA1c is also described, also mainly in woman. HbA1c and T1D were closely related in the management and diagnosis of the disease. HbA1c is a blood test that reflects the average blood glucose levels over the past 2–3 months and it measures the percentage of hemoglobin that is glycated. Therefore, HbA1c is the cornerstone of managing T1D, offering a window into how well blood sugar has been controlled over time. It helps guide treatment adjustments and predict risk for complications.

SCFAs are fatty acids with fewer than six carbon atoms that are primarily produced by the fermentation of dietary fibers by gut microbiota in the colon. In recent years, different articles have been published analyzing SCFA in T1D, but only a few of them have analyzed circulating SCFA in human samples. A previous study in 53 T1D patients and 50 HC analyzing AA, PA, and BA in plasma and feces using liquid chromatography–mass spectrometry described that plasma levels of AA and PA were lower in T1D, with similar fecal SCFA results [18]. In a recent paper with 198 adults T1D patients, but without HC, serum SCFA levels and blood glucose control were assessed by HbA1c and continuous glucose monitoring metrics, and dietary intake from a 7-day food record; authors found that SCFA levels showed significant sex-specific differences and a sex-specific association between serum PA levels and blood glucose control in women with T1D [19]. SCFA levels have also been measured in urine samples collected from 98 children (40 HC, 40 T1D, and 18 obese children): PA, IBA, BA VA levels were higher in T1D patients than HC [20]. These contradictory results could be due to the low number of samples analyzed in some of these studies, the different origin of the samples, the different techniques used, the different SCFA analyzed and also the heterogeneous cohorts of T1D patients recruited in these studies. A systematic review and meta-analysis aimed to assess the effect of SCFA interventions on fasting glucose, fasting insulin, and homeostatic model assessment of insulin resistance (HOMA-IR) has been recently published. The authors concluded that those studies with a confirmed increase in SCFAs at the end of intervention also had a significant effect on lowering fasting insulin, and elevated levels of SCFAs, compared with baseline levels, were associated with beneficial effects on HOMA-IR. However, in this meta-analysis they did not differentiate between the different SCFA [21]. Therefore, it is difficult to establish a conclusion comparing our results with those previously published. However, an association between SCFA levels and HbA1c, glucose, and/or fasting insulin seem to be plausible. Finally, our study showed differences in SCFA levels in men and women. These sex-specific differences have been found by other authors, as we have shown above [19]. Furthermore, a previous publication analyzing sex-specific changes in the gut microbiome and host metabolome of T1D mice via 16S rRNA gene sequencing and nuclear magnetic resonance-based metabolomics approach suggests that the sex-dependent gut microbiota–host metabolism axis may be implicated in the sexual dimorphism of T1D [22].

In this study, as we have performed similarly in previous studies with multiple sclerosis patients, we have calculated the ratio between AA and the other SCFA. In previous studies, we suggested that AA could have a pro-inflammatory role, while, on the contrary, PA and BA would have an anti-inflammatory one [23]. The ratio not only gives us information about the potential imbalance between the AA and the other SCFA, but also allows us to correct the individual heterogeneity in SCFA levels due to individual factors like different gut bacterial populations or gut permeability. As in MS, in this study we also found lower ratios in T1D patients, showing that the SCFA imbalance could be a common mechanism in autoimmune diseases, and restoring this equilibrium could be essential for the control of these pathologies.

Furthermore, in our study, a higher proportion of AA was associated with a lower number of SCFA detected. A bidirectional feedback loop exists between inflammation and the gut microbiota, particularly involving bacteria that produce inflammatory metabolites. During inflammation, the gut environment changes, favoring the growth of certain bacteria. These bacteria produce pro-inflammatory compounds which can damage the intestinal barrier and activate immune receptors. This immune activation leads to the release of cytokines, further fueling inflammation. The ongoing inflammatory state promotes dysbiosis. A dysbiotic microbial community, once established, substantially affects both the local mucosal and systemic landscape of immune cells, thereby creating a feedback loop in which the host immune system and its microbiota cross-regulate each other [24]. These bacteria, in turn, continue to produce metabolites that worsen inflammation and disrupt gut homeostasis. This self-reinforcing cycle plays a critical role in chronic conditions like inflammatory disorders. Breaking this loop is a key therapeutic goal, often approached through dietary interventions [25], probiotics [26], or fecal microbiota transplantation [27]. Understanding this dynamic interplay is essential for developing strategies to restore gut health and immune balance.

The other metabolite that was found in association with different clinical and analytical variables in T1D patients was p-cresol. Furthermore, T1Dm showed higher levels of p-cresol than HCm and a positive correlation between p-cresol and age was found both in T1Dw and T1Dm. p-cresol is a microbial metabolite produced in the gut from the fermentation of aromatic amino acids, primarily tyrosine. It is a phenolic compound with significant implications for host physiology, especially in the context of gut health, liver metabolism, and systemic effects [28]. In our study, p-cresol was positively correlated with ferritin levels. Ferritin is a blood protein that stores iron, and its levels are often used to assess iron status in the body. However, ferritin is also an acute-phase reactant, meaning it can be elevated in the presence of inflammation, infection, or liver disease, even when iron stores are normal or low [29]. Thus, we found that p-cresol levels were lower in T1D patients with a normal liver profile than in T1D patients with a not normal liver profile (7.5 μM vs. 13.0 μM, respectively; *p* = 0.002). After production in the gut, p-cresol is absorbed and sulfated or glucuronidated in the liver, forming p-Cresyl sulfate or p-Cresyl glucuronide (pCG). Therefore, higher levels of p-cresol could be associated with disturbances in normal liver function in T1D patients. Since T1Dm showed higher levels of p-cresol than HCm, this metabolite could be associated with sex differences in liver disturbances among individuals with T1D, although this should be deeper analyzed in future studies. Finally, p-cresol levels were lower in T1D patients without high blood pressure than in T1D patients with high blood pressure (6.7 μM vs. 10.6 μM, respectively; *p* = 0.005). High blood pressure (hypertension) is a common and serious comorbidity in individuals with T1D, significantly increasing the risk of cardiovascular disease, kidney damage, and other diabetes-related complications. The development of hypertension in people with T1D is often linked to diabetic nephropathy, where persistent hyperglycemia leads to kidney damage, further elevating blood pressure and creating a harmful feedback loop [30]. Furthermore, there is a significant relationship between liver disturbances, hypertension, and T1D, primarily through mechanisms involving insulin resistance, metabolic dysfunction, and systemic inflammation [31], often linked to metabolic syndrome components like obesity/overweight [32]. Elevated serum p-cresol levels have been observed in individuals with obesity, indicating a potential link between this metabolite and adiposity. A study involving 373 patients with stable coronary artery disease found significant positive correlations between serum levels of total para-cresylsulfate (a metabolite of p-cresol) and central obesity indices such as waist-to-hip ratio, conicity index, and body shape index. These associations were particularly pronounced in male patients, suggesting a sex-specific relationship between para-cresylsulfate levels and central obesity [33]. Furthermore, a systematic review of metabolomics studies highlighted that obese individuals often exhibit higher concentrations of p-cresol and its sulfate conjugate in urine, indicating increased production of these metabolites [34]. Therefore, p-cresol levels could be a biomarker for these comorbidities in T1D patients. Strategies directed to reduce overweight and to control liver disturbances and hypertension could be monitored through the analysis of the p-cresol levels in the serum of the T1D patients. The positive correlation found between p-cresol and age in both, male and female, could be finally associated with the increasing percentage of these comorbidities with the age in T1D patients. Further studies are guaranteed to explain the associations here described for the p-cresol.

However, a limitation of the study could be the lack of information about the diet of patients and controls, since diet can modify the composition of the microbiota, and therefore, the circulating metabolites, as has been previously shown with the AA levels [19]. Furthermore, although we have not found any association between the duration of T1D and the levels and ratios of the different gut metabolites analyzed, it could be very interesting to analyze these data since the beginning of the disease, and not only in cohort with a well-established disease. Finally, a proportion of circulating SCFA levels may derive from endogenous processes such as acetogenesis; since endogenous production of SCFAs may be increased in patients with T1D, particularly those with poor glycemic control, this mechanism could partly explain the observed association between SCFA levels and HbA1c levels.

In conclusion, bacterial metabolite levels were altered in T1D patients. AA levels were significantly increased in T1D patients, mainly in T1D women, and AA levels positively correlated with HbA1c, also mainly in woman. PA/AA and BA/AA ratios were lower in T1D patients, showing that the SCFA imbalance could be a common mechanism in autoimmune diseases. Finally, p-cresol levels correlated with ferritin levels and they were higher in T1D patients with liver disturbances and hypertension. As has been previously suggested by other authors [25,35], developing strategies to restore gut health and immune balance could be useful for the control of T1D.

## 4. Materials and Methods

### 4.1. Study Design

We performed an observational prospective cross-sectional study, including samples from Type 1 Diabetes (T1D) patients and healthy controls (HC). T1D patients were recruited from the Endocrinology Service of the Hospital Clínico San Carlos between 2024 and 2025. A cohort of HC with a similar sex and age distribution were recruited among the volunteered blood donors of Hospital Clínico San Carlos between 2024 and 2025. Demographic and clinical data are shown in Table 2. Categorical and continuous variables collected from T1D patients are shown in Table 3.

### 4.2. Ethics Statement

Informed consent was obtained from all T1D and HC involved in the study. This study was approved by the local Ethic Committee of the Hospital Clínico San Carlos (Comité Ético de Investigación Clínica del Hospital Clínico San Carlos), with the following approval number: C.I. 22/002-E. The study was conducted in accordance with the Declaration of Helsinki.

### 4.3. Sample Collection

Whole blood was obtained by venipuncture from every T1D patient and HC. Blood was located in a Leucosep tube pre-filled with Leucosep separation medium (Greiner Bio-One, Kremsmünster, Germany). PBMCs and plasma were isolated following manufacturer instructions. Plasma was aliquoted in 0.2 ml tubes and stored at −80 °C until use.

### 4.4. SCFA, MCFA, and p-Cresol Levels Determination

Aliquots of plasma were processed as previously described by CGEM service at IRYCIS: https://www.irycis.org/es/servicios/45/cromatografia-de-gases-acoplada-a-espectrometria-de-masas (accesed on 3 February 2025) [36]. Briefly, at least 100 μL of plasma was extracted with 300 μL mixture composed of 299.5 μL ethanol and 0.5 μL of deuterated butyric acid D7 4 g/L. After vigorous vortex and pipetting (10 min), the samples were centrifuged for 10 min at 13,000× *g*. The supernatant was transferred to a new tube with 5 μL of freshly prepared 0.8 M sodium hydroxide. Solvents were evaporated using a vacuum centrifuge to completely dryness (Thermo Fisher SpeedVac™ SPD121P, Dreieich, Germany). The residual salts were redissolved in 100 μL of a mixture of 6:1 ratio of ethyl alcohol and acidified with 0.6 M succinic acid immediately before the analysis. Gas chromatography–mass spectroscopy (GC-MS) analysis was performed using a TRACE 1600/1610 gas chromatograph/ISQ7610 mass selective detector (Thermo Fisher Scientific, Dreieich, Germany) equipped with a TG-WaxMS A GC Column (30 m × 0.25 mm × 0.25 μm, Thermo Fisher Scientific, Dreieich, Germany). The injector, GC-MS transfer line and ion source temperature were set to 200 °C, 215 °C, and 250 °C, respectively. The flow rate of the helium carrier gas was set at 1 mL/min. We then introduced 1 μL of the sample by splitless injection. The initial column temperature was set to 55 °C and held for 1 min, then raised to 105 °C at a rate of 8 °C/min where it was held for 2 min. Lastly, the column temperature was raised to 190 °C at a rate of 30 °C/min and kept at this temperature for 1 min. An extra step was added to delete possible leftovers, the temperature was raised to 210 °C at a rate of 20 °C/min and held at this temperature for 3 min. The ionization was performed in the electron impact mode at 70 eV. The MS data were acquired by SIM scan mode, scan spectra were collected at a scan time 0.5 min. The compounds were identified by comparing the obtained MS spectra to the National Institute of Standards and Technology database and confirmed by comparing to the retention times of pure standards. The instrument was operated, and the data acquired and analyzed using Chromeleon 7 software. The bacterial metabolites identified and quantified are shown in Table 4.

### 4.5. Statistical Analysis

Categorical variables were expressed as percentages, normal numerical variables as mean ± standard deviation, and non-normal as median (25th, 75th percentile). The association between/among categorical variables was analyzed using the Chi-square χ^2^ test, or Fisher’s exact test when the value of the expected count less than 5 is more than 20%. For the quantitative variables, the means were compared using Student’s *t*-test or analysis of variance or the Mann–Whitney U test, in case the quantitative variables did not fit a normal distribution. The parametric Pearson coefficient or the nonparametric Spearman coefficient was applied to evaluate the correlation between two continuous quantitative variables. Since a correlation coefficient < 0.3 or >−0.3 could be considered as negligible correlation [37], we only considered those above or below these values, respectively. Subjects with missing data were omitted from the corresponding analyses. *p*-values < 0.05 were referred to as statistically significant in the text. When necessary, the Bonferroni adjustment was carried out. All analyses were conducted using SPSS for Windows (Ver. 21.0) software (SPSS Inc., Chicago, IL, USA)), and plots were elaborated with Prism version 8.0 (GraphPad Prism, San Diego, CA, USA).

## Figures and Tables

**Table 1:** Levels and ratios of SCFA and levels of MCFA and p-crestol in T1D patients and HC by sex.

	HCw *	HCm *	*p* **	T1Dw *	T1Dm *	*p* **	*p* * HCw vs. T1Dw	*p* * HCm vs. T1Dm
**AA (μg/mL)**	249.2	296.5	n.s.	316.1	317.4	n.s.	0.003	n.s.
**PA (μg/mL)**	12.4	12.0	n.s.	12.8	12.5	n.s.	n.s.	n.s.
**IBA (μg/mL)**	6.8	6.1	n.s.	6.4	6.2	n.s.	n.s.	n.s.
**BA (μg/mL)**	3.2	2.7	n.s.	3.3	3.2	n.s.	n.s.	n.s.
**IVA (μg/mL)**	11.5	12.9	n.s.	12.8	12.2	n.s.	n.s.	n.s.
**VA (μg/mL)**	3.1	1.8	n.s.	1.9	2.7	n.s.	n.s.	n.s.
**MVA (μg/mL)**	66.2	56.4	n.s.	69.5	53.7	n.s.	n.s.	n.s.
**PA/AA**	0.052	0.040	0.009	0.036	0.039	n.s.	0.00008	n.s.
**IBA/AA**	0.029	0.022	0.026	0.019	0.019	n.s.	0.0002	n.s.
**BA/AA**	0.014	0.010	0.042	0.012	0.011	n.s.	0.041	n.s.
**IVA/AA**	0.052	0.040	n.s.	0.049	0.038	n.s.	n.s.	n.s.
**VA/AA**	0.016	0.007	n.s.	0.007	0.007	n.s.	n.s.	n.s.
**MVA/AA**	0.257	0.170	0.027	0.223	0.158	n.s.	n.s.	n.s.
**HxA (μg/mL)**	5.6	6.1	n.s.	5.5	5.7	n.s.	n.s.	n.s.
**HpA (μg/mL)**	44.3	11.2	n.s.	6.5	2.5	n.s.	0.027	n.s.
**p** **-cresol (μg/mL)**	8.1	6.2	0.039	7.3	8.5	n.s.	n.s.	0.022

* median values. ** *p*-values were calculated using Student’s *t*-test; n.s.: not significant.

**Table 2:** Clinical and demographic data.

	T1D (n = 91)	HC (n = 58)	*p*
**Sex (female/n)**	51/91	31/58	n.s.
**Age at sampling (years, m ± SD)**	47.6 ± 13.5	46.2 ± 11.8	n.s.
**Age at T1D onset (years, m ± SD)**	19.9 ± 12.1	-	-
**T1D duration (months, md (P25-P75))**	354 (206–450)	-	-
**% HbA1c at sampling (md (P25-P75))**	7.1% (6.7–7.7%)	-	-
% T1D patients with serious complications *	45/91 (49.5%)	-	-
% T1D patients with other diagnostics **	16/91 (17.6%)	-	-

* As serious complications of T1D were considered: microvascular complications (retinopathy, microalbuminuria, peripheral neuropathy, and ischemic heart disease) and acute hypoglycemia episodes. ** Other diagnostics: celiac disease, psoriasis, rheumatoid arthritis, asthma, vitiligo, papillary thyroid carcinoma, ovarian carcinoma, erosive gastritis, chronic gastritis, irritable bowel syndrome, Addison’s disease, grave’s disease, and epilepsy.

**Table 3:** Clinical and analytical variables collected in T1D patients.

Categorical Variables	Continuous Variables
Sex (female; male)	Age
Debut	Age at diagnosis
Current treatment (CSII; MDI)	Disease duration
Hidroferol treatment (yes; no)	Weight
Serious complications (yes; no)	Body Mass Index
Other diagnostics (yes; no)	% HbA1c
High Blood Pressure (yes; no)	Glucose
Hypercholesterolemia (yes; no)	Vitamin D
Hypothyroidism (yes; no)	Vitamin B12
Liver profile (normal; not normal)	Creatinine
Glomerular filtration rate (>90%/<90%)	Albumin
	Folic Acid
	Ferritin
	C-Reactive Protein

**Table 4:** Bacterial metabolites analyzed by GC-MS.

	Name	Abbreviation	N° of carbons
Short-chain fatty acids:	Acetate	AA	2
	Propionate	PA	3
	Butyrate	BA	4
	Isobutyrate	IBA	4
	Methyl valerate	MVA	5
	Isovalerate	IVA	5
	Valerate	VA	5
Medium-chain fatty acids:	Hexanoate or Caproate	HxA	6
	Heptanoate or Enanthate	HpA	7
Aromatic compounds:	Para-cresol	p-cresol	7

## Data Availability

The original contributions presented in the study are included in the article. Further inquiries can be directed to the corresponding author.
